# Correlates of neural adaptation to food cues and taste: the role of obesity risk factors

**DOI:** 10.1093/scan/nsab018

**Published:** 2021-03-03

**Authors:** Jennifer R Sadler, Grace E Shearrer, Afroditi Papantoni, Sonja T Yokum, Eric Stice, Kyle S Burger

**Affiliations:** Department of Nutrition, Gillings School of Global Public Health, University of North Carolina at Chapel Hill, Chapel Hill, NC 27516, USA; Department of Family and Consumer Sciences, College of Agriculture, University of Wyoming, Laramie, WY 82071, USA; Department of Nutrition, Gillings School of Global Public Health, University of North Carolina at Chapel Hill, Chapel Hill, NC 27516, USA; Oregon Research Institute, Eugene, OR 97403, USA; Department of Psychiatry and Behavioral Sciences, Stanford University Medical Center, Stanford, CA 94304, USA; Department of Nutrition, Gillings School of Global Public Health, University of North Carolina at Chapel Hill, Chapel Hill, NC 27516, USA; Biomedical Research Imaging Center, University of North Carolina at Chapel Hill, Chapel Hill, NC 27516, USA

**Keywords:** fMRI, incentive sensitization, obesity, adolescent, taste

## Abstract

Identifying correlates of brain response to food cues and taste provides critical information on individual differences that may influence variability in eating behavior. However, a few studies examine how brain response changes over repeated exposures and the individual factors that are associated with these changes. Using functional magnetic resonance imaging, we examined how brain response to a palatable taste and proceeding cues changed over repeated exposures and how individual differences in weight, familial obesity risk, dietary restraint and reward responsiveness correlate with these changes. In healthy-weight adolescents (*n* = 154), caudate and posterior cingulate cortex (PCC) response increased with repeated cue presentations, and oral somatosensory cortex and insula response increased with repeated milkshake tastes. The magnitude of increase over exposures in the left PCC to cues was positively associated with body mass index percentile (*r* = 0.18, *P* = 0.026) and negatively associated with dietary restraint scores (*r* = −0.24, *P* = 0.003). Adolescents with familial obesity risk showed higher cue-evoked caudate response across time, compared to the low-risk group (*r* = 0.12, *P* = 0.035). Reward responsiveness positively correlated with right oral somatosensory cortex/insula response to milkshake over time (*r* = 0.19, *P* = 0.018). The results show that neural responses to food cues and taste change over time and that individual differences related to weight gain are correlated with these changes.

## Introduction

Maintaining energy balance is a primary drive and is achieved through various means of balancing energetic intake with output (Keesey and Powley, 2008). A key component of this system is motivation; palatable foods have rewarding post-ingestive effects that promote further consumption of the food through associative learning (Sclafani, 2001). Both humans and animals learn to associate visual cues for a palatable food with the food’s motivational value over repeated exposures (Johnson, 2013; Davidson *et al.*, 2014). While preference for foods (e.g. liking) is mostly stable, desire for a food or its motivational value is more sensitive to state-dependent changes (Finlayson *et al.*, 2007; Berridge *et al.*, 2009). One example of a state that impacts food wanting is sensory-specific satiety (SSS). SSS is the decline in desire to consume a food further as the food is eaten. SSS does not represent absolute satiation, since following SSS, the desire to consume foods with different sensory properties (e.g. taste) remains the same (Havermans, 2012). This decline in pleasantness during consumption can render a preferred food unpalatable. Sensitivity to SSS is proadaptive in the modern food environment, where large portions of highly palatable foods are readily available and encourage individuals to overconsume (Ello-Martin *et al.*, 2005), which may result in unhealthy weight gain.

Multiple systems are theorized to drive SSS, including those implicated in reinforcement learning. Response habituation, the process by which response diminishes with repeated exposure to a stimulus, is a leading theory for the mechanism of SSS (Epstein *et al.*, 2009). Also, stimulus specificity is demonstrated in SSS (Havermans, 2012), meaning that hedonic decline, or the loss of pleasantness, occurs for only the food that is consumed to satiation. Habituation and stimulus sensitivity are key components of reinforcement learning, suggesting SSS may represent a form of appetitive conditioning. In animals, SSS decreases the dopaminergic signal in regions of the midbrain and prefrontal cortex, including the nucleus accumbens, orbitofrontal cortex (OFC) and medial prefrontal cortex (Ahn and Phillips, 1999; O’Doherty *et al.*, 2000). In human neuroimaging research, SSS is linked to decreased response in the OFC following repeated exposure to a food odor, relative to an unexposed food odor (O’Doherty *et al.*, 2000). These regions are also important for reinforcement learning, further supporting the idea that SSS and appetitive conditioning may act through similar pathways.

Repeated exposure to a cue and paired reward is also associated with the development of incentive sensitization. Following cue–reward pairings, the motivational value of the cue increases, and desire for the reward is transferred to the cue (Robinson and Berridge, 1993). This wanting, called incentive sensitization, remains even when the primary reward is no longer reinforcing (Robinson and Berridge, 1993). In eating behavior, incentive sensitization explains how individuals can become highly responsive to palatable food cues, resulting in food cravings and cued overeating, even in the absence of hunger (Robinson and Berridge, 1993). Greater striatal response to food cues reflects the development of incentive sensitization (Flagel *et al.*, 2011; Burger and Stice, 2014). Incentive sensitization and SSS represent different adaptations that change the motivational value of foods and their cues. In the brain, incentive sensitization increases the motivational response to a cue, while SSS can dampen the value signal for a taste over time. However, the neural changes reflecting these adaptive processes have not been investigated within the same paradigm.

Individual differences in brain response during SSS and incentive sensitization to food cues may explain variation in eating behaviors including meal initiation and meal termination. Identifying these factors may improve the ability to predict unhealthy weight gain/obesity risk. Consumption of a high-fat, high-sugar diet diminishes SSS in animals (Reichelt *et al.*, 2014). In humans, obesity has been correlated with lower sensitivity to SSS (Epstein *et al.*, 1996); however, the effect is inconsistent (Brondel *et al.*, 2007). Obesity is associated with other eating behavior dysregulations, which may confound the relationship between obesity and SSS. Therefore, it may be useful to evaluate individual differences in SSS in healthy-weight individuals. Food cue responsivity is also correlated with obesity, where individuals at an elevated weight or with familial risk of obesity show increased response to food cues in striatal and midbrain regions (Stice *et al.*, 2011; Yokum *et al.*, 2011). Finally, psychological constructs are related to cue responsiveness. In one study, trait reward responsiveness was related to elevated attention to food cues and greater response in the striatum, amygdala and OFC (Beaver *et al.*, 2006).

To address gaps in the literature around correlates of brain adaptation to food cues and taste, we tested how whole-brain response to palatable milkshake receipt and milkshake cue changed with repeated exposure to the stimuli. Early exposure was compared to late exposure, and the magnitude of brain response to cue/milkshake receipt was then correlated with individual-level factors related to eating behavior, including parental obesity, body mass index (BMI), BMI percentile, percent body fat, dietary restraint and disinhibition, and reward and punishment responsiveness. We hypothesized that repeated exposure to the milkshake cue would correlate with increased response in the striatum, and repeated exposure to milkshake would correlate with decreased response in OFC. We hypothesized that BMI and weight gain risk factors (e.g. parental weight status and high dietary restraint scores) would be related to greater magnitude of the hypothesized changes.

## Methods

### Sample recruitment

Data for this analysis were collected as part of a broader study to examine how obesity risk determinants affect brain response to anticipation and receipt of a palatable beverage and the relation of brain response to future weight change in adolescents (Stice *et al.*, 2011; Stice *et al.*, 2015). Data were collected from July 2009 to May 2011 in Eugene, Oregon, USA. Participants were recruited through a number of methods including advertisements in local newspapers, Craigslist postings, flyers posted in local schools and word-of-mouth referrals. Participants were screened for eligibility by research personnel via a phone interview. Eligibility criteria included the following: (i) aged 14–17 years, (ii) BMI between 18.0 and 25.0 kg/m^2^ and (iii) either two parents with BMIs in overweight/obese range (≥25.0 kg/m^2^) or two parents with BMIs in normal-weight range (18.5–25.0 kg/m^2^). Exclusion criteria were as follows: (i) current use of psychoactive medications or illicit drugs more than weekly, (ii) contraindication of magnetic resonance imaging (MRI), such as pregnancy and recent head injury with a loss of consciousness, (iii) significant cognitive impairment, (iv) any major medical problems such as type I diabetes, (v) diagnosis of current Axis I psychiatric disorder and (vi) one parent with BMI in overweight/obese range and one parent with BMI in normal range. For eligible participants, parental and participant consent was obtained before data collection. All methods and procedures were approved by the Institutional Review Board of Oregon Research Institute.

### Study overview

Data collection included two baseline assessments: (i) a behavioral assessment and (ii) a functional neuroimaging scanning assessment. At the behavioral assessment, participants completed demographic measures (including age, race/ethnicity, biological sex and parental education), metabolic and physiological measures including height and weight measurements and a Bod-Pod assessment of body composition. Participants also completed questionnaires assessing eating behavior and reward sensitivity. During the behavioral assessment, parents provided self-reported height and weight data for themselves and the adolescent’s other biological parent. At the neuroimaging assessment, participants completed internal state and perceptual hedonic ratings of tastants and a functional MRI (fMRI) scan including the food reward task. Participants were asked to consume typical meals the day of their scanning assessment, but refrain from eating or drinking (except water) for 4–6 h before the assessment. The majority of participants completed the paradigm between 16:00 and 18:00 h (∼5 h after eating a typical lunch). Full details regarding the broader study from which this sample is drawn can be found elsewhere (Stice *et al.*, 2011; Stice and Yokum, 2016).

### Measures

#### BMI, BMI percentile and familial risk for obesity.

Participants’ height and weight were measured at baseline using an electronic scale and stadiometer. Height was measured to the nearest millimeter, and weight was assessed to the nearest 0.1 kg after removal of shoes and coats. Two measures of each were obtained and averaged. BMI and BMI-for-age percentile, which accounts for a child/adolescent’s age and biological sex, were calculated in accordance with US Centers for Disease Control growth charts (Kuczmarski *et al.*, 2000). Familial risk for obesity was assessed using parental BMI. Parental height and weight were self-reported and then verbally confirmed for both biological parents with the parent who attended the assessment. From this, parental BMI was calculated. The high-weight-gain risk group comprised adolescents with two parents with overweight or obesity (BMI > 25.0 kg/m^2^), and adolescents with two parents at normal weight were categorized as low-weight-gain risk.

#### Body composition.

Body composition was assessed using air displacement plethysmography via the Bod-Pod (CosMed, Rome, Italy). Participants were instructed to wear snug swimsuits and a swim cap for the assessment to minimize trapped air mass. Two measurements were performed for each participant, and the results were averaged. Body density was calculated as body mass (assessed by direct weighing) divided by estimated body volume. Percent body fat was estimated from body density.

#### Eating behavior and reward sensitivity questionnaires.

Eating behavior was assessed using the Three-Factor Eating Questionnaire (TFEQ; Stunkard and Messick, 1985). We calculated scores on two of the questionnaire’s three scales assessing different constructs: dietary disinhibition and dietary restraint. Each scale has shown internal consistency (α = 0.90 and 0.92; Stunkard and Messick, 1985). Scores on the TFEQ predict future weight change (James *et al.*, 2017). Non-specific reward/punishment sensitivity was assessed using the Behavioral Inhibition Scale and Behavioral Approach Scale (BIS/BAS; Carver and White, 1994). The scales measure motivation to avoid punishment (BIS) and motivation to approach rewarding outcomes (BAS). Scores on the BIS/BAS correlate with 30% of the variation in BMI in men and women (Dietrich *et al.*, 2014) and correlate with weight change in men (Mussap, 2006). The scales have good internal consistency (BIS: α = 0.74; BAS: α = 0.72 Carver and White, 1994).

#### Tastant liking, wanting and internal state ratings.

Participants rated their hunger and the perceived pleasantness of and desire to consume the milkshake and tasteless solution before and after the fMRI scan. Ratings were completed using 20 cm cross-modal visual analog scales (VASs). VAS ratings were anchored by 0 (‘not at all’), 10 (‘neutral’) and 20 (‘never been more hungry’). Ratings were converted to 0–100 scale by dividing the rating by the total length of the scale.

#### Substance use and negative consequences questionnaire.

Illicit drug use was measured using a self-report questionnaire assessing alcohol and drug use and negative consequences. Participants reported the frequency of alcohol and drug use in the past year. Additionally, participants reported the frequency of negative consequences from drug/alcohol use in the past year.

#### fMRI food reward task.

The food reward fMRI paradigm examines response to milkshake receipt and milkshake cues. Participants were instructed to eat regularly on the day of their scan, but to refrain from eating or drinking for 4–6 h immediately preceding their scan. Participants completed five runs of a task where they received tastes of a chocolate milkshake and tasteless solution designed to mimic the osmolarity of saliva. The milkshake consisted of 2 scoops of vanilla ice cream, 1.5 cups of 2% milk and 2 tablespoons of chocolate syrup, representing a palatable, high-fat and high-sugar beverage (Stice *et al.*, 2011). The tasteless solution consisted of 25 mm KCl and 2.5 mm NaHCO_3_ in distilled water (O’Doherty *et al.*, 2001). Stimuli were presented in the five runs in a randomized order.

**Fig. 1.**
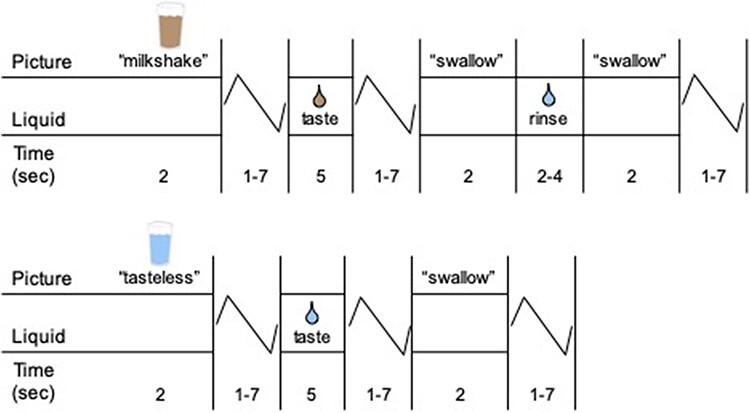
fMRI food reward paradigm.

Participants were presented with cues consisting of images of a glass of milkshake or water that signaled the delivery of 0.5 ml of a chocolate milkshake or a tasteless solution, respectively. However, on 40% of the trials, the corresponding taste was not delivered following its cue. Cues preceding taste were categorized as ‘paired’ cues, while cues that did not precede taste were categorized as ‘unpaired’ cues. Cues were presented for 2 s and were followed by a jitter of 1–7 s, during which time the screen was blank. Taste delivery lasted 5 s. Participants were instructed to swallow when they saw the ‘swallow’ cue. A 0.5 ml of rinse delivered over 2–4 s followed milkshake receipt. The next trial began 1–7 s after the ‘swallow’ cue went off (Figure 1). Tastes were delivered using programmable syringe pumps to ensure consistent volume, rate and timing of taste delivery. Sixty milliliter syringes filled with milkshake and tasteless solution were connected via Tygon tubing (B-44-3; inner diameter: 3/32 inch; outer diameter: 5/32 inch; 50 ft length) through a wave guide to a manifold attached to the sliding table of the scanner. The manifold fit into the participants’ mouths and delivered the taste to a consistent segment of the tongue.

Timing of milkshake cue and receipt and tasteless solution cue–receipt trials in the food reward paradigm.

### Neuroimaging data parameters and data preprocessing

Scanning was performed by a Siemens Allegra 3 Tesla head-only MRI scanner (Siemens Medical Solutions, Munich, Germany). A birdcage coil acquired data from the entire brain. A thermo-foam vacuum pillow and additional padding restricted head motion. Functional scans used a T2*-weighted gradient single-shot echo planar imaging sequence [echo time, 30 ms; repetition time (TR), 2000 ms; flip angle, 80°] with an in-plane resolution of 3.0 × 3.0 mm^2^ [64 × 64 matrix; field of view (FOV), 192 × 192 mm^2^]. To cover the whole brain, thirty-two 4 mm slices (interleaved acquisition, no skip) were acquired along the anterior commissure–posterior commissure transverse, oblique plane, as determined by the midsagittal section. Prospective acquisition correction was applied to adjust slice position and orientation, as well as to re-grid residual volume-to-volume motion in real time during data acquisition for the purpose of reducing motion-induced effects (Thesen *et al.*, 2000). Structural scans were collected using an inversion recovery T1-weighted sequence (MP-RAGE) in the same orientation as the functional sequences to provide detailed anatomic images aligned to the functional scans. High-resolution structural MRI sequences (FOV, 256 × 256 mm^2^; 256 × 256 matrix; thickness, 1.0 mm; slice number ≈ 160) were acquired.

Imaging data were preprocessed and analyzed using SPM software package (Version 12, Wellcome Department of Imaging Neuroscience, London, UK) in MATLAB (MathWorks, Natick, MA, USA) (Worsley and Friston, 1995). Images were time-acquisition corrected to the slice obtained at 50% of the TR. Functional images were then realigned to the mean. Images (anatomical and functional) were normalized to the standard Montreal Neurological Institute (MNI) template brain. Normalization resulted in a voxel size of 3 mm^3^ for functional images and a voxel size of 1 mm^3^ for structural images. Functional images were smoothed with a 6 mm full-width half maximum isotropic Gaussian kernel. Functional data were assessed to detect spikes in global mean response and motion outliers in the functional data using the Artifact Detection Toolbox (Gabrieli Lab, McGovern Institute for Brain Research, Cambridge, MA). Motion parameters were included as regressors in the design matrix at individual-level analysis. Additionally, image volumes where the *z*-normalized global brain activation exceeded 3 s.d.s from the mean of the run or showed greater than 1 mm of composite (linear plus rotational) movement were flagged as outliers and deweighted during individual-level model estimation. Analyses with and without the inclusion of motion artifacts did not impact the presented results.

To identify response to milkshake cues, blood oxygen level–dependent (BOLD) response during presentation of the milkshake cue was contrasted against the tasteless solution cue (milkshake cue > tasteless solution cue). No difference in BOLD response was observed between the paired *vs* unpaired cues for the milkshake and tasteless solutions, so cues were collapsed into the same condition. To identify response to milkshake receipt, BOLD response during milkshake receipt was contrasted against tasteless solution receipt (milkshake receipt > tasteless solution receipt). Vectors of the onsets for each event of interest were compiled and convolved by the canonical hemodynamic response function. Additional covariates of no interest included the time of the swallow cue, motion parameters and temporal derivatives of the hemodynamic function. A 128 s high-pass filter was used to remove low-frequency noise and slow drifts in the signal.

To model main effects of the paradigm, participants’ five runs of task data were included in a random-effects model to identify average response during the two contrasts of interest: milkshake cue > tasteless cue and milkshake receipt > tasteless receipt. To model the differences in brain response during early *vs* late exposures, two first-level models were generated for each subject: (i) an early task phase (early exposure) model consisting of the first two runs (totaling 12 events of each taste and 20 events of each cue) and (ii) a late task phase (late exposure) model consisting of the final three runs (totaling 18 events of each taste and 30 events of each cue). Early/late exposures were selected to include as many events as possible in each condition, without splitting data collected in the same run. At the group level, the resulting images for early and late exposures were entered into a within-subjects paired sample *t*-test comparing early *vs* late response in the milkshake cue > tasteless cue contrast and milkshake receipt > tasteless receipt contrast. For main effects and repeated-exposure effects, the T-map threshold was set at SPM-derived family-wise error corrected *P*_FWE_ < 0.05 and a cluster size of 10 voxels (Nichols and Hayasaka, 2003). Significant clusters are reported with cluster size (*k*), cluster-level *P*_FWE_ value, peak *z*-statistic and peak coordinates in MNI space.

The resulting significant clusters from early *vs* late models were saved as regions of interest (ROIs). Average parameter estimates (PEs) within the ROIs for clusters were extracted from each participant’s data using the MarsBaR toolbox (Version 0.44) in SPM12. This generated subject-specific PEs for early exposure and late exposure in each contrast of interest (cue and taste) in the ROIs. The magnitude of change from early to late exposure was calculated as the difference between PEs.

### Behavioral data analysis

Statistical analyses of behavioral data and PEs were carried out using the R statistical software package (version 3.5.1, R Foundation for Statistical Computing, Vienna, Austria). Descriptive statistics including mean and s.d. were computed for all behavioral variables. Paired samples *t*-tests were used to test for significant changes in VAS scores from pre- to post-scan. To test for biological and behavioral factors associated with the magnitude of PE change in significant ROIs, Pearson’s product-moment correlation tests were carried out for the following variables: BMI, BMI percentile, %body fat, TFEQ disinhibition and restraint scores, and BIS/BAS scores. Repeated-measures analysis of variance (ANOVA) tests were used to examine differences in early–late PEs between familial obesity risk groups. Time was modeled as a within-subject factor, and familial obesity risk was modeled as a between-subject factor. Repeated-measures ANOVAs included an error term to account for between-subject variation in change over time. Significant between-subject effects identified on repeated-measures ANOVA were verified using independent samples *t*-tests with mean PEs in ROIs. Significance was considered at an uncorrected statistical threshold of two-tailed *P* < 0.05. Effect sizes are presented to provide insight into the robustness of effects, and the Benjamini–Hochberg procedure was applied to adjust for multiple comparisons (p-FDR (p-false discovery rate)) (Benjamini and Hochberg, 1995).

## Results

### Sample characteristics

One hundred and sixty-two healthy-weight adolescents completed baseline assessments. From the sample, 154 adolescents were included in the present analyses [2 were excluded due to bad segmentation of the anatomical (used in registration), 4 due to incomplete neuroimaging data collection (e.g. >2 runs where acquisition cut short/missing due to discomfort/having to use the restroom) and 2 were excluded for errors in field map acquisition]. The analytic sample was on average 15.2 years of age, healthy-weight, non-Hispanic and white (Table 1). From pre- to post-scan, there was a significant decrease in milkshake wanting (*t* = 20.0, *P *< 0.001) and liking ratings (*t* = 5.4, *P* < 0.001) on VASs. There was also a significant increase in hunger from pre- to post-scan (*t*= −6.6, *P* < 0.001). Over two-thirds (*n* = 105; 68.2%) of the sample reported no drug/alcohol use in the past year. Twenty-six percent (*n* = 40; 26%) of the sample reported any drug/alcohol use within the past year, and nine participants (5.8%) reported substance use at least once per month in the past year (Table 1). Eleven participants (7.1%) reported experiencing a negative consequence of drug/alcohol use in the past year, with four (2.7%) of participants reporting two or more negative consequences in the past year.

**Table 1.** Participant characteristics (*n* = 154)

Characteristics	Count (frequency)
Gender	
Female	78 (50.6%)
Male	76 (49.4%)
Race	
American Indian or Alaska Native	3 (1.9%)
Asian	1 (0.6%)
Black or African American	0 (0%)
Native Hawaiian or Pacific Islander	0 (0%)
White	130 (84.4%)
More than one race	12 (7.8%)
Other	8 (5.2%)
Ethnicity	
Hispanic	18 (11.7%)
Non-Hispanic	134 (87.0%)
Missing	1 (0.6%)
Weight gain risk status	
High risk	118 (76.6%)
Low risk	36 (23.4%)
Substance use	
Never in past year	105 (68.2%)
At least once in past year	40 (26.0%)
Monthly in past year	9 (5.8%)
Parental education: father	
Grade school	4 (2.7%)
Some high school	18 (12.0%)
High school degree	24 (16.0%)
Some college	34 (22.7%)
College degree	48 (32.0%)
Advanced degree	21 (14.0%)
Missing	1 (0.7%)
Parental education: mother	
Grade school	2 (1.3%)
Some high school	7 (4.6%)
High school degree	24 (15.8%)
Some college	43 (28.3%)
College degree	55 (36.2%)
Advanced degree	20 (13.2%)
Missing	1 (0.7%)
Mean ± s.d.	
Age (years)	15.2 ± 2.02
BMI (kg/m^2^)	20.9 ± 1.94
BMI percentile	53.6 ± 21.7
TFEQ—restraint (0–20)	5.9 ± 2.85
TFEQ—disinhibition (0–16)	3.7 ± 1.93
TFEQ—hunger (0–15)	5.5 ± 2.78
BIS (0–4)	2.8 ± 0.58
BAS (0–4)	3.0 ± 0.41
VAS milkshake ratings (0–100)	
Liking—pre-scan	80.0 ± 14.7
Liking—post-scan^**^	72.1 ± 16.2
Wanting—pre-scan	77.4 ± 13.6
Wanting—post-scan^**^	37.3 ± 19.6
VAS hunger ratings (0–100)	
Hunger—pre-scan	39.6 ± 23.0
Hunger—post-scan^**^	57.5 ± 23.3

** Significant change in ratings from pre- to post-scan (*P* value <0.001).

### Main effects of brain response to fMRI food reward paradigm

**Fig. 2.**
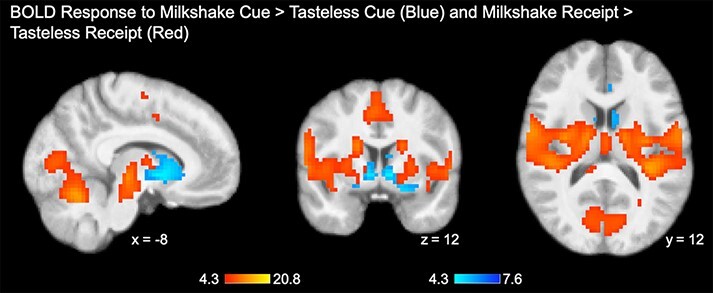
Main effects of BOLD response to milkshake cue and milkshake receipt.

In response to the milkshake cue (contrasted with the tasteless cue), elevated BOLD response was observed in three clusters (Figure 2, in blue): a large cluster spanning bilateral caudate (peak = 9, 15, 3; *Z* = 7.02; *k *= 543; *P*_FWE_ < 0.001), a cluster in the occipital fusiform cortex (peak = −21, −84, −12; *Z* = 5.85; *k *= 12; *P*_FWE_ = 0.001) and a cluster in the anterior cingulate cortex (peak = −3, 30, 18; *Z* = 5.29; *k *= 50; *P*_FWE_ < 0.001). Robust activity was observed in response to milkshake receipt compared to tasteless solution receipt across six clusters (Figure 2, in red). The largest cluster shows peaks throughout the bilateral pre/postcentral gyrus (peak = 42, −12, 33; *Z* > 8; *k *= 4754; *P*_FWE_ < 0.001). Other clusters include response in the left VI- lobule of the cerebellum and intracalcarine cortex (peak = −15, −63, −21; *Z* > 8; *k *= 945; *P*_FWE_ < 0.001), the supplementary motor cortex (peak = 3, −6, 63; *Z* > 8; *k *= 232; *P*_FWE_ < 0.001), the precuneus (peak= 27, −45, 18; *Z* > 8; *k *= 25; *P*_FWE_ < 0.001), and additional pre/postcentral clusters in the right (peak = 21, −27, 60; *Z* > 8; *k *= 41; *P*_FWE_ < 0.001) and left hemispheres (peak = −21, −30, 60; *Z* > 8; *k *= 39; *P*_FWE_ < 0.001).

Main effects of milkshake cue (>tasteless cue) are shown in blue. Significant response was observed in the bilateral caudate (peak = 9, 15, 3; *Z* = 7.02; *k *= 543; *P*_FWE_ < 0.001), the occipital fusiform cortex (peak = −21, −84, −12; *Z* = 5.85; *k *= 12; *P*_FWE_ = 0.001) and the anterior cingulate cortex (peak = −3, 30, 18; *Z* = 5.29; *k *= 50; *P*_FWE_ < 0.001). Response to milkshake receipt (>tasteless receipt) is shown in red. Significant clusters were found in left and right pre/postcentral gyrus (peak = 42, −12, 33; *Z* > 8; *k *= 4754; *P*_FWE_ < 0.001), the left VI of the cerebellum and intracalcarine cortex (peak = −15, −63, −21; *Z* > 8; *k *= 945; *P*_FWE_ < 0.001), the supplementary motor cortex (peak = 3, −6, 63; *Z* > 8; *k *= 232; *P*_FWE_ < 0.001), the precuneus (peak= 27, −45, 18; *Z* > 8; *k *= 25; *P*_FWE_ < 0.001) and two additionally pre/postcentral clusters: right (peak = 21, −27, 60; *Z* > 8; *k *= 41; *P*_FWE_ < 0.001) and left (peak = −21, −30, 60; *Z* > 8; *k *= 39; *P*_FWE_ < 0.001).

### Differences in brain response as a function of repeated exposures

**Fig. 3.**
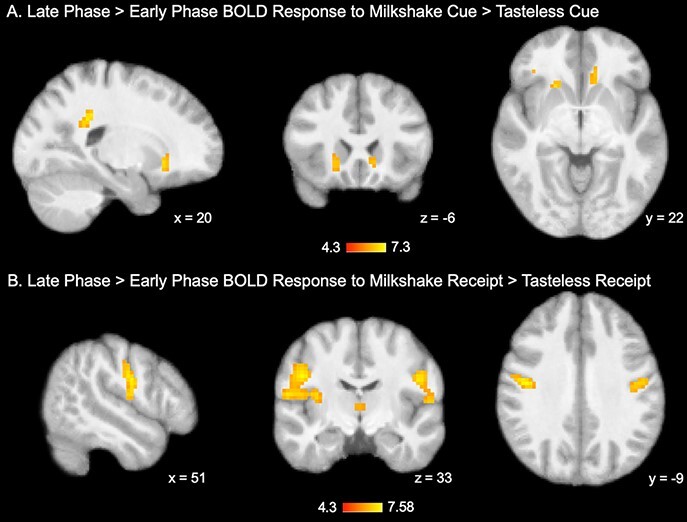
Differences in BOLD response to milkshake cues and receipt as a function of repeated exposures.

When contrasting late exposure (runs 3–5) with early exposure (runs 1 and 2), elevated BOLD response to milkshake cue and milkshake receipt was found (Figure 3; Table 2). Regions that showed an increased response over repeated cue exposures included four clusters: two clusters representing the bilateral posterior cingulate cortex (PCC), extending into white matter, and in two clusters representing the bilateral caudate (Figure 3; Table 2). In response to milkshake receipt (compared to tasteless solution receipt), increased BOLD response over repeated exposures was observed in two clusters containing bilateral oral somatosensory cortex (pre/postcentral gyrus) extending into the dorsal insula (Figure 3; Table 2). There was no significant response observed in the early exposure > late exposure contrast.

**Table 2.** Greater BOLD response as a function of repeated exposures (late exposure relative to early exposure)

Milkshake cue > tasteless cue	*x* ^a^	*y*	*z*	*k* ^b^	*Z*-value	*P*_FWE_
Left posterior cingulate cortex (PCC)	−15	−45	21	45	6.75	<0.001
Precuneus	−18	−42	36		6.14	<0.001
Posterior cingulate cortex (PCC)	−15	−42	12		4.89	0.014
Right posterior cingulate cortex (PCC)	24	−39	33	40	6.34	<0.001
Right putamen and caudate	21	21	−6	18	5.86	<0.001
Left caudate	−12	30	−6	20	5.32	0.002
Milkshake receipt > tasteless receipt						
Right postcentral gyrus	45	−12	33	184	6.98	<0.001
Right precentral gyrus	54	−3	24		6.92	<0.001
Right insula	36	−9	15		6.32	<0.001
Left postcentral gyrus	−48	−9	27	112	6.91	<0.001
Left central operculum	−57	−12	9		6.44	<0.001
Left precentral/postcentral gyrus	−45	−15	36		6.30	<0.001

a MNI coordinates (mm).

b Cluster size.

(A) Late exposure (runs 3–5) contrasted against early exposure (runs 1 and 2) showed elevated BOLD response to milkshake cue (>tasteless cue) in the posterior cingulate cortex (left: −15, −45, 21; *k *= 45; *P*_FWE_ < 0.001; right: 24, −39, 33; *k *= 40, *P*_FWE_ < 0.001) and caudate (left: −12, 30, −6; *k *= 20; *P*_FWE_ = 0.002; right: 21, 21, −6; *k *= 18, *P*_FWE_ < 0.001). (B) Late exposure (>early exposure) to milkshake receipt (>tasteless receipt) was associated with greater BOLD response in the bilateral oral somatosensory cortex extending into the dorsal insula (left: −48, −9, 27; *k *= 112; *P*_FWE_ < 0.001; right: 45, −12, 33; *k *= 184, *P*_FWE_ < 0.001).

### Correlates of brain response change over repeated exposures

**Fig. 4.**
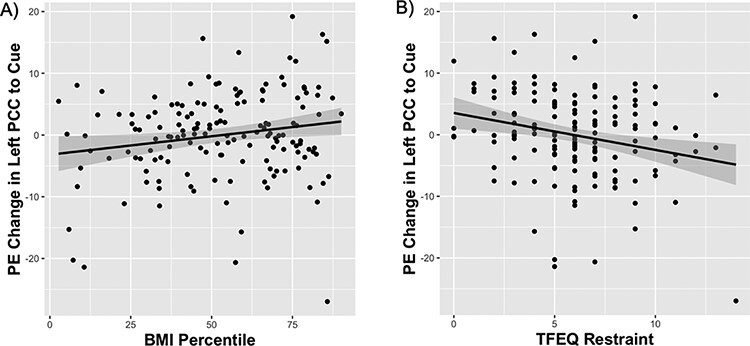
BMI percentile and TFEQ restraint scores are associated with change in the left posterior cingulate cortex response.

**Fig. 5.**
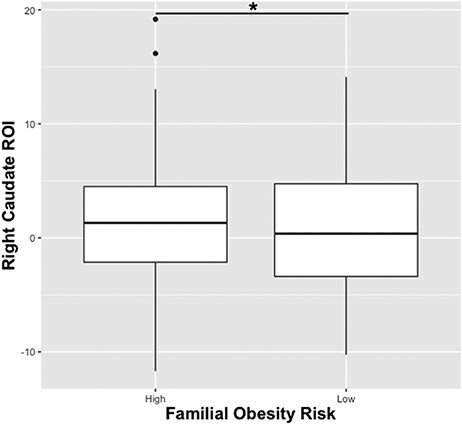
Familial obesity risk is associated with significantly higher right caudate response.

BMI percentile was positively correlated with response in the left PCC over repeated exposures to milkshake cues (*r *= 0.18, *t* = 2.28, *P*_uncorrected_ = 0.024; p-FDR = 0.12; Figure 4A), while TFEQ dietary restraint subscale scores were negatively associated with the change in the left PCC (*r* = −0.24, *t* = −3.05, *P*_uncorrected_ = 0.003; p-FDR = 0.055; Figure 4B). Parental obesity group (both parents with overweight or obese *vs* both parents with normal BMI) was associated with milkshake cue response in the right caudate independent of time (*F*(1302) = 4.15; eta-squared = 0.014; *P*_uncorrected_ = 0.0425; p-FDR = 0.38; Figure 5). An exploratory, independent samples *t*-test of mean right caudate response between the maternal and paternal BMI groups supported a significant effect of paternal BMI group on caudate response (*t* = −2.65, df = 160.12, *P* value = 0.0089). While adolescents at high risk for weight gain showed a slight decrease in right caudate response over time and adolescents at low risk showed an increase over time in the same region, the interaction of parental obesity and time was not significant (*F*(1302) = 0.151; *P*_uncorrected_ = 0.70).

(A) BMI percentile is positively correlated with response in the left PCC over repeated exposures to milkshake cues (*r* = 0.18, *t* = 2.28, *P* = 0.024). (B) TFEQ restraint subscale scores were negatively associated with the change in the left PCC (*r* = −0.24, *t* = −3.05, *P* = 0.003).

Familial obesity was associated with the magnitude of milkshake cue response in the right caudate (*t* = −2.65, df = 160.12, *P* = 0.0089) with stronger right caudate response among participants with familial obesity risk (two parents with BMI >25.0 kg/m^2^) *vs* participants without familial obesity risk (two parents with BMI <25.0 kg/m^2^).

In exploratory analyses, we found that the between-subject factor of paternal overweight/obesity (compared to paternal normal weight) was associated with stronger right caudate response to milkshake cue, independent of time (*F*(1300) = 7.55; eta-squared = 0.025; *P*_uncorrected_ = 0.0064), but there was no significant interaction of parental overweight/obesity and time (*F*(1300) = 0.003; *P*_uncorrected_ = 95). An exploratory independent samples *t*-test of mean right caudate response between the paternal BMI groups (healthy BMI *vs* overweight/obese BMI) supported a significant effect of paternal BMI group on caudate response (*t* = −2.41, df = 161.2, *P* value = 0.017). There was no between-subjects effect of maternal overweight/obesity on right caudate response (*F*(1302) = 0.31; *P*_uncorrected_ = 0.58), and the interaction of maternal overweight/obesity and time was not associated with right caudate response (*F*(1302) = 1.40; *P*_uncorrected_ = 0.24).

Other measures of adiposity (BMI, %body fat) and constructs associated with weight regulation (dietary disinhibition, hunger and BIS) were not correlated with the degree of change as a function of repeated exposures in the ROIs (*P*s = 0.07–0.97). Further, the magnitude of change in VAS ratings for milkshake liking/wanting from pre-scan to post-scan was not correlated to the degree of change as a function of repeated exposures in the ROIs (*P*s = 0.08–0.98). In an exploratory analysis, there were no differences in brain response between participants who reported no drug use, any drug use in the past year or monthly drug use in the past year (*P*s = 0.30–0.99) or any differences in response by parental education (*P*s = 0.27–0.97). Full results are presented in Supplemental Materials.

## Discussion

Over the course of eating, incentive sensitization and SSS affect food cue responsiveness and motivation to consume the same food further. How sensitive individuals are to these adaptations and how brain response changes over consumption have been theorized to explain some level of individual variation in eating behavior. Individual differences in adaptations have been connected to physiological and behavioral characteristics such as weight, weight gain risk conferred by genetics or parental obesity, and eating behavior constructs (Epstein *et al.*, 1996; Stice *et al.*, 2011; Rapuano *et al.*, 2017). The present study builds on this work, demonstrating that repeated exposure to a milkshake cue was associated with greater response in the caudate and PCC over time, and repeated administration of a milkshake was associated with a greater response in the oral somatosensory cortex and dorsal insula. Perceptual hedonic ratings showed significant decreases in milkshake liking and wanting following repeated exposures. Further, individual differences in neural change over time were observed. The cue-evoked change in the left PCC was correlated positively with BMI percentile and negatively with dietary restraint. BMI percentile is a risk factor for future obesity (Guo *et al.*, 2002), while dietary restraint shows inconsistent relationship with weight change across studies (Lowe *et al.*, 2013). Also, cue-evoked signal in the right caudate was stronger in adolescents at high risk *vs* low risk for future obesity as determined by parental BMI, suggesting that high-risk adolescents may have increased motivational susceptibility to food cues. Finally, the magnitude of change to milkshake receipt was positively correlated with trait reward responsiveness. Together, these results suggest that brain response adapts to repeated milkshake cue and taste exposure in regions important for gustatory processing and motivation and that the degree of change is sensitive to individual differences in adiposity and weight gain risk factors.

Across the sample, brain response to milkshake cue and receipt was seen in a number of regions associated with motivation, visual processing and behavior anticipation and modulation. The observed response is similar to prior research testing brain response to cue-elicited anticipation of a palatable taste and receipt of the taste (O’Doherty *et al.*, 2002; Stice *et al.*, 2008). The brain adaptation to milkshake cues reported here is also similar to prior studies of cue–reinforcer pairing. The striatum, inclusive of the caudate, is key for associative learning from primary and secondary reinforcers (O’Doherty *et al.*, 2004; Delgado *et al.*, 2005; Camara *et al.*, 2008), and cue-evoked response in the caudate is shown to increase over time when paired with taste reinforcement (Schultz, 1998; Burger and Stice, 2014). This caudate adaptation may reflect increased dopamine response to a predictive cue, which is seen following repeated cue–reward exposure (Robinson and Berridge, 1993; Richardson and Gratton, 1996; Schultz, 1998). Increased BOLD response to the food cue was also found in the PCC, although the cluster reported is partially localized in white matter. The PCC is involved in arousal, attention and integrating information across the brain (Leech and Sharp, 2014), and PCC response is thought to help adapt behavior to optimize outcomes (Pearson *et al.*, 2011). Further, PCC response increases with the reward value of food cues (Litt *et al.*, 2011; García-García *et al.*, 2013), and PCC response to food cues is higher in individuals with obesity compared to those with normal BMI (Rothemund *et al.*, 2007). Caudate signaling increases with incentive sensitization (Flagel *et al.*, 2011), and given the PCC is responsive to rewarding food cues, the brain adaptions to repeated cue exposure reported here may reflect incentive sensitization to the milkshake cue.

Adaptations in cue-evoked BOLD response were correlated with body weight, obesity risk and dietary restraint. While results did not survive adjustment for multiple comparisons, the uncorrected relationships are considered. The magnitude of change over time in the left PCC was positively correlated with BMI percentile and negatively correlated with dietary restraint. Elevated response in the PCC to food cues has been previously associated with obesity (Flagel *et al.*, 2011), and obesity is associated with an increased bias toward food cues in a sated state (Cornier *et al.*, 2009; Nummenmaa *et al.*, 2012). Given that PCC response is thought to reflect increased attention and behavioral adaption, its positive correlation with BMI percentile suggests that healthy-weight adolescents at a higher BMI percentile may be more attuned to the milkshake cues. This could contribute to a more rapid incentive sensitization in higher BMI percentile adolescents who are otherwise healthy (Castellanos *et al.*, 2009). Conversely, dietary restraint was inversely associated with cue-evoked PCC adaptation. Individuals with high dietary restraint are highly sensitive to food cues (Fedoroff *et al.*, 2003; Talepasand and Golzari, 2018), but successful restrained eaters may engage top-down control over food cue responsiveness (Johnson *et al.*, 2012; Alblas *et al.*, 2020). The association of dietary restraint with dampened PCC adaptation may reflect attempts to control attention to food cues in high restraint participants. Finally, familial obesity risk conferred by parental overweight/obesity was associated with stronger cue-evoked response in the right caudate. The differential response observed between risk groups is echoed in other research. In a similar, but independent sample, adolescents at risk for obesity show elevated response in the caudate to milkshake receipt (Shearrer *et al.*, 2018). In another sample, elevated cue-evoked caudate response over conditioning was associated with future weight gain (Burger and Stice, 2014), and in the same sample using an ROI-based approach focused on striatal response, adolescents at high risk for obesity conferred by parental obesity were found to have a higher caudate response to food receipt (Burger and Stice, 2011). Despite a slight decrease with repeated exposure, caudate response in the high-risk group was higher than that of the low-risk group. Higher caudate response to milkshake cues suggests that high-risk adolescents may be more motivated by food cues early into the task or may have a greater susceptibility to incentive sensitization to food cues. Increased cue responsiveness could explain how elevated BMI percentile and parental obesity increase adolescents’ risk of onset and maintenance of overweight or obesity in adulthood. Prior research in this sample found that stronger baseline striatal response to milkshake cues predicted body fat gain over 3 years of follow-up (Stice and Yokum, 2016), suggesting that higher caudate responses in adolescents with familial obesity risk may have implications for change in body composition over time. Additionally, the effect of parental BMI on cue-evoked response in the right caudate varied between maternal and paternal obesity. In an exploratory analysis, we found that paternal obesity was significantly positively associated with right caudate response, while maternal obesity showed no relation. This result is unexpected, as previous research found that maternal obesity had a stronger influence than paternal obesity on weight gain risk in children (Whitaker *et al.*, 2010). In consideration of these results, it is important to note that parental BMI was self-reported, and one biological parent reported the height and weight for the other biological parent. Misreporting could affect parental BMI values and confound the results. Despite this limitation, these results may suggest that paternal BMI has a stronger influence on brain response to food cues, while maternal BMI has a greater influence on weight gain.

In response to milkshake receipt, we found that repeated exposures to milkshake were associated with greater BOLD response in the bilateral pre/postcentral gyrus and dorsal insula. The pre/postcentral gyrus represents the oral somatosensory area and classically responds strongly to beverage administration (Miyamoto *et al.*, 2006). BOLD responses in the oral somatosensory cortex and anterior insula are associated with gustatory processing of mouthfeel, taste and thermal properties of tastes (Small, 2010; Iannilli *et al.*, 2014). Conversely, the dorsal insula is thought to be more closely related to cognitive control and executive function (Uddin *et al.*, 2017). The observed increased response in the oral somatosensory cortex and dorsal insula after repeated taste exposure is unexpected; we hypothesized that repeated milkshake administration would relate to decreased OFC response, reflecting SSS. In this sample, VAS ratings of milkshake liking and wanting significantly decreased following the scan, which in prior studies was observed following SSS (Havermans *et al.*, 2009). However, fMRI response to the milkshake did not show the expected neural signature of SSS. While participants reported a significant decrease in wanting ratings, suggesting SSS, the observed decrease in wanting was not related to the magnitude of change in brain response to milkshake taste from early exposure to late exposure. A possible interpretation for this discrepancy is that the present results represent an earlier stage of neural adaptation to SSS. During this task, participants received a comparatively small amount of milkshake (0.5 ml per trial, total 15 ml over the scan). Physiological/hormonal responses that contribute to SSS may not have been elicited by the small volume of milkshake consumed (Kadohisa, 2015). A disconnect between subjective self-report measures and implicit measures is a noted challenge in eating behavior research, as self-reported appetite ratings do not reliably map onto behavior (Holt *et al.,* 2017). It is possible that the conflicting results reported here may be subject to this effect.

Alternatively, the present results may be unrelated to SSS and instead reflect increased somatosensory response over time. Further research into the dynamics of neural adaptations to taste is needed to better understand the present results. Notably, the magnitude of change in brain response to milkshake cues and receipt in the six ROIs were not associated with perceptual hedonic ratings of the milkshake. The lack of association between neural adaptation to milkshake cue/taste exposure and measures of tastant liking and wanting similarly raises questions about what brain adaptation represents, since brain adaptation in the OFC is linked to the decline in wanting in satiety (Jiang *et al.*, 2015). It is likely that the adaptation to milkshake receipt found in our sample reflects a process outside of SSS.

This study had several limitations. First, while we observed associations between the change in brain response over time with physiological and behavioral characteristics of the sample, significant associations did not survive adjustment for multiple comparisons using the false discovery rate. Second, the sample is predominantly white and non-Hispanic. While the sample is representative of the region from which it was drawn, our results are not generalizable to the broader US population. It is critical that future research recruits diverse samples to increase the impact of findings. Third, possible confounding factors including the recency of drug use were not collected in the sample. Recent drug use can affect motivation/reward systems in the brain (Koob and Le Moal, 2001) and may impact the present results. Finally, to maximize power and limit the impact of trial-by-trial noise in cue and milkshake response, we, a priori, selected cut points to define the first two runs of the task as early exposure and the last three runs as late exposure. However, this approach is not sensitive to possible individual differences in the onset of incentive sensitization or SSS (Flagel *et al.*, 2007). One possible analytic method to address this limitation is the application of dynamic connectivity regression (DCR) to taste administration fMRI data (Cribben *et al.*, 2012). This method identifies state-related changes in brain networks through the use of functional connectivity. The network structure changes over repeated exposures to a cue and reward (Gerraty *et al.*, 2018), so this method could be applied to identify individual differences in the onset and the number of network state changes during repeated exposure to a milkshake cue or taste. This would provide a data-driven method for identifying individual differences in sensitivity to incentive sensitization or SSS and could be applied to improve obesity risk assessments in adolescents. Of note, DCR is best implemented in blocked paradigms. Lastly, we elected the sample from the early and late runs, given the anticipated effect size and the adequate amount of data as we believed that this was the most sensitive approach; however, a parametric approach to modeling the change over time could also be applied. Future research in how brain response changes across cue/taste exposure may benefit from changing taste administration paradigms from event-related designs to blocked designs to allow for DCR methods or applying parametric approaches to understand how brain response changes over time.

Behavioral adaptations to repeated food cue and taste exposure are well documented; however, a few studies have examined how BOLD response changes over this process. In a large, adolescent sample, we found that brain changes occur with repeated exposure to a milkshake cue and taste in regions important for motivation, attention and awareness, and oral sensation and executive function. Echoing studies of static brain response to food cues and taste, we found that individual characteristics such as adiposity, familial obesity risk and reward responsiveness amplified adaptation, while dietary restraint dampened the degree of change over time. The results indicate that individual differences in obesity risk factors affect how the brain adapts to food cues over time, possibly increasing incentive sensitization and providing a mechanism for future risk of overeating and obesity. As the present sample comprised of healthy-weight adolescents, our findings can be used to identify at-risk adolescents for obesity prevention programs.

## Supplementary Material

nsab018_Supp

## References

[R1] Ahn, S., Phillips, A.G. (1999). Dopaminergic correlates of sensory-specific satiety in the medial prefrontal cortex and nucleus accumbens of the rat. *The Journal of Neuroscience*, 19(19), RC29. 10.1523/JNEUROSCI.19-19-j0003.1999 PMC6782999 10493774

[R2] Alblas, M.C., Mollen, S., Fransen, M.L., Van Den Putte, B. (2020). Food at first sight: an eye-tracking study on visual attention to palatable food cues on TV and subsequent unhealthy food intake in unsuccessful dieters. *Appetite*, 147, 104574.doi: 10.1016/j.appet.2019.104574. 31877342

[R3] Beaver, J.D., Lawrence, A.D., Van Ditzhuijzen, J., Davis, M.H., Woods, A., Calder, A.J. (2006). Individual differences in reward drive predict neural responses to images of food. *The Journal of Neuroscience*, 26(19), 5160–6. doi: 10.1523/JNEUROSCI.0350-06.2006. 16687507 PMC6674259

[R4] Benjamini, Y., Hochberg, Y. (1995). Controlling the false discovery rate: a practical and powerful approach to multiple testing. *Journal of the Royal Statistical Society: Series B (Methodological)*, 57(1), 289–300. doi: 10.1111/j.2517-6161.1995.tb02031.x.

[R5] Berridge, K.C., Robinson, T.E., Aldridge, J.W. (2009). Dissecting components of reward: ‘liking’, ‘wanting’, and learning. *Current Opinion in Pharmacology*, 9(1), 65–73. doi: 10.1016/j.coph.2008.12.014. 19162544 PMC2756052

[R6] Brondel, L., Romer, M., Van Wymelbeke, V., et al. (2007). Sensory-specific satiety with simple foods in humans: no influence of BMI? *International Journal of Obesity*, 31(6), 987–95. doi: 10.1038/sj.ijo.0803504. 17160089

[R7] Burger, K.S., Stice, E. (2014). Greater striatopallidal adaptive coding during cue-reward learning and food reward habituation predict future weight gain. *Neuroimage*, 99, 122–8. doi: 10.1016/j.neuroimage.2014.05.066. 24893320 PMC4142439

[R8] Camara, E., Rodriguez-Fornells, A., Münte, T.F. (2008). Functional connectivity of reward processing in the brain. *Frontiers in Human Neuroscience*, 2, 19. doi: 10.3389/neuro.09.019.2008. PMC2647336 19242558

[R9] Carver, C.S., White, T.L. (1994). Behavioral inhibition, behavioral activation, and affective responses to impending reward and punishment: the BIS/BAS scales. *Journal of Personality and Social Psychology*, 67(2), 319–33. doi: 10.1037/0022-3514.67.2.319.

[R10] Castellanos, E.H., Charboneau, E., Dietrich, M.S., et al. (2009). Obese adults have visual attention bias for food cue images: evidence for altered reward system function. *International Journal of Obesity*, 33(9), 1063–73. doi: 10.1038/ijo.2009.138. 19621020

[R11] Cornier, M.-A., Salzberg, A.K., Endly, D.C., Bessesen, D.H., Rojas, D.C., Tregellas, J.R. (2009). The effects of overfeeding on the neuronal response to visual food cues in thin and reduced-obese individuals. *PLoS One*, 4(7), e6310. doi: 10.1371/journal.pone.0006310. PMC2712682 19636426

[R12] Cribben, I., Haraldsdottir, R., Atlas, L.Y., Wager, T.D., Lindquist, M.A. (2012). Dynamic connectivity regression: determining state-related changes in brain connectivity. *Neuroimage*, 61(4), 907–20. doi: 10.1016/j.neuroimage.2012.03.070. 22484408 PMC4074207

[R13] Davidson, T.L., Sample, C.H., Swithers, S.E. (2014). An application of Pavlovian principles to the problems of obesity and cognitive decline. *Neurobiology of Learning and Memory*, 108, 172–84. doi: 10.1016/j.nlm.2013.07.014. 23887140 PMC3899105

[R14] Delgado, M.R., Miller, M.M., Inati, S., Phelps, E.A. (2005). An fMRI study of reward-related probability learning. *Neuroimage*, 24(3), 862–73. doi: 10.1016/j.neuroimage.2004.10.002. 15652321

[R15] Dietrich, A., Federbusch, M., Grellmann, C., Villringer, A., Horstmann, A. (2014). Body weight status, eating behavior, sensitivity to reward/punishment, and gender: relationships and interdependencies. *Frontiers in Psychology*, 5, 1073. doi: 10.3389/fpsyg.2014.01073. PMC4202791 25368586

[R16] Ello-Martin, J.A., Ledikwe, J.H., Rolls, B.J. (2005). The influence of food portion size and energy density on energy intake: implications for weight management. *The American Journal of Clinical Nutrition*, 82(1 Suppl), 236S–41S. doi: 10.1093/ajcn/82.1.236S. 16002828

[R17] Epstein, L.H., Temple, J.L., Roemmich, J.N., Bouton, M.E. (2009). Habituation as a determinant of human food intake. *Psychological Review*, 116(2), 384–407. doi: 10.1037/a0015074. 19348547 PMC2703585

[R18] Epstein, L.H., Paluch, R., Coleman, K.J. (1996). Differences in salivation to repeated food cues in obese and nonobese women. *Psychosomatic Medicine*, 58(2), 160–4. doi: 10.1097/00006842-199603000-00011. 8849634

[R19] Fedoroff, I., Polivy, J., Herman, C.P. (2003). The specificity of restrained versus unrestrained eaters’ responses to food cues: general desire to eat, or craving for the cued food? *Appetite*, 41(1), 7–13. doi: 10.1016/s0195-6663(03)00026-6. 12880616

[R20] Finlayson, G., King, N., Blundell, J.E. (2007). Liking vs. wanting food: importance for human appetite control and weight regulation. *Neuroscience and Biobehavioral Reviews*, 31(7), 987–1002. doi: 10.1016/j.neubiorev.2007.03.004. 17559933

[R21] Flagel, S.B., Cameron, C.M., Pickup, K.N., Watson, S.J., Akil, H., Robinson, T.E. (2011). A food predictive cue must be attributed with incentive salience for it to induce c-fos mRNA expression in cortico-striatal-thalamic brain regions. *Neuroscience*, 196, 80–96. doi: 10.1016/j.neuroscience.2011.09.004. 21945724 PMC3206316

[R22] Flagel, S.B., Watson, S.J., Robinson, T.E., Akil, H. (2007). Individual differences in the propensity to approach signals vs goals promote different adaptations in the dopamine system of rats. *Psychopharmacology*, 191(3), 599–607. doi: 10.1007/s00213-006-0535-8. 16972103

[R23] García-García, I., Narberhaus, A., Marqués-Iturria, I., et al. (2013). Neural responses to visual food cues: insights from functional magnetic resonance imaging. *European Eating Disorders Review*, 21(2), 89–98. doi: 10.1002/erv.2216. 23348964

[R24] Gerraty, R.T., Davidow, J.Y., Foerde, K., Galvan, A., Bassett, D.S., Shohamy, D. (2018). Dynamic flexibility in striatal-cortical circuits supports reinforcement learning. *The Journal of Neuroscience*, 38(10), 2442–53. doi: 10.1523/JNEUROSCI.2084-17.2018. 29431652 PMC5858591

[R25] Guo, S.S., Wu, W., Chumlea, W.C., Roche, A.F. (2002). Predicting overweight and obesity in adulthood from body mass index values in childhood and adolescence. *The American Journal of Clinical Nutrition*, 76(3), 653–8. doi: 10.1093/ajcn/76.3.653. 12198014

[R26] Havermans, R.C., Janssen, T., Giesen, J.C.A.H., Roefs, A., Jansen, A. (2009). Food liking, food wanting, and sensory-specific satiety. *Appetite*, 52(1), 222–5. doi: 10.1016/j.appet.2008.09.020. 18951934

[R27] Havermans, R.C. (2012). Stimulus specificity but no dishabituation of sensory-specific satiety. *Appetite*, 58(3), 852–5. doi: 10.1016/j.appet.2012.02.009. 22343169

[R0027a] Holt, G. M., Owen, L. J., Till, S., Cheng, Y., Grant, V. A., Harden, C. J., and Corfe, B. M., (2017). Systematic literature review shows that appetite rating does not predict energy intake. *Critical reviews in food science and nutrition*, 57(16), 3577–3582. doi: 10.1016/j.appet.2012.02.009. 27736161

[R28] Iannilli, E., Noennig, N., Hummel, T., Schoenfeld, A.M. (2014). Spatio-temporal correlates of taste processing in the human primary gustatory cortex. *Neuroscience*, 273, 92–9. doi: 10.1016/j.neuroscience.2014.05.017. 24846613

[R29] James, B.L., Loken, E., Roe, L.S., Rolls, B.J. (2017). The Weight-Related Eating Questionnaire offers a concise alternative to the Three-Factor Eating Questionnaire for measuring eating behaviors related to weight loss. *Appetite*, 116, 108–14. doi: 10.1016/j.appet.2017.04.023. 28442337 PMC5520533

[R30] Jiang, T., Soussignan, R., Schaal, B., Royet, J.-P. (2015). Reward for food odors: an fMRI study of liking and wanting as a function of metabolic state and BMI. *Social Cognitive and Affective Neuroscience*, 10(4), 561–8. doi: 10.1093/scan/nsu086. 24948157 PMC4381239

[R31] Johnson, A.W. (2013). Eating beyond metabolic need: how environmental cues influence feeding behavior. *Trends in Neurosciences*, 36(2), 101–9. doi: 10.1016/j.tins.2013.01.002. 23333343

[R32] Johnson, F., Pratt, M., Wardle, J. (2012). Dietary restraint and self-regulation in eating behavior. *International Journal of Obesity*, 36(5), 665–74. doi: 10.1038/ijo.2011.156. 21829162

[R33] Kadohisa, M. (2015). Beyond flavour to the gut and back. *Flavour*, 4(1), 1–11. doi: 10.1186/s13411-015-0047-8.

[R34] Keesey, R.E., Powley, T.L. (2008). Body energy homeostasis. *Appetite*, 51(3), 442–5. doi: 10.1016/j.appet.2008.06.009. 18647629 PMC2605663

[R35] Koob, G.F., Le Moal, M. (2001). Drug addiction, dysregulation of reward, and allostasis. *Neuropsychopharmacology*, 24(2), 97–129. doi: 10.1016/S0893-133X(00)00195-0. 11120394

[R36] Kuczmarski, R.J., Ogden, C.L., Grummer-Strawn, L.M., et al. (2000). CDC growth charts: United States. *Advance Data*, 314, 1–27. 11183293

[R37] Leech, R., Sharp, D.J. (2014). The role of the posterior cingulate cortex in cognition and disease. *Brain: A Journal of Neurology*, 137(Pt 1), 12–32. doi: 10.1093/brain/awt162. 23869106 PMC3891440

[R38] Litt, A., Plassmann, H., Shiv, B., Rangel, A. (2011). Dissociating valuation and saliency signals during decision-making. *Cerebral Cortex*, 21(1), 95–102. doi: 10.1093/cercor/bhaq065. 20444840

[R39] Lowe, M.R., Doshi, S.D., Katterman, S.N., Feig, E.H. (2013). Dieting and restrained eating as prospective predictors of weight gain. *Frontiers in Psychology*, 4, 577. doi: 10.3389/fpsyg.2013.00577. PMC3759019 24032024

[R41] Miyamoto, J.J., Honda, M., Saito, D.N., et al. (2006). The representation of the human oral area in the somatosensory cortex: a functional MRI study. *Cerebral Cortex*, 16(5), 669–75. doi: 10.1093/cercor/bhj012. 16079244

[R42] Mussap, A.J. (2006). Reinforcement sensitivity theory (RST) and body change behaviour in males. *Personality and Individual Differences*, 40(4), 841–52. doi: 10.1016/j.paid.2005.08.013.

[R43] Nichols, T., Hayasaka, S. (2003). Controlling the familywise error rate in functional neuroimaging: a comparative review. *Statistical Methods in Medical Research*, 12(5), 419–46. doi: 10.1191/0962280203sm341ra. 14599004

[R44] Nummenmaa, L., Hirvonen, J., Hannukainen, J.C., et al. (2012). Dorsal striatum and its limbic connectivity mediate abnormal anticipatory reward processing in obesity. *PLoS One*, 7(2), e31089. doi: 10.1371/journal.pone.0031089. PMC3272045 22319604

[R45] O’Doherty, J., Rolls, E.T., Francis, S., et al. (2000). Sensory-specific satiety-related olfactory activation of the human orbitofrontal cortex. *Neuroreport*, 11(2), 399–403. doi: 10.1097/00001756-200002070-00035. 10674494

[R46] O’Doherty, J., Rolls, E.T., Francis, S., Bowtell, R., McGlone, F. (2001). Representation of pleasant and aversive taste in the human brain. *Journal of Neurophysiology*, 85(3), 1315–21. doi: 10.1152/jn.2001.85.3.1315. 11248000

[R47] O’Doherty, J., Dayan, P., Schultz, J., Deichmann, R., Friston, K., Dolan, R.J. (2004). Dissociable roles of ventral and dorsal striatum in instrumental conditioning. *Science*, 304(5669), 452–4. doi: 10.1126/science.1094285. 15087550

[R48] O’Doherty, J.P., Deichmann, R., Critchley, H.D., Dolan, R.J. (2002). Neural responses during anticipation of a primary taste reward. *Neuron*, 33(5), 815–26. doi: 10.1016/s0896-6273(02)00603-7. 11879657

[R49] Pearson, J.M., Heilbronner, S.R., Barack, D.L., Hayden, B.Y., Platt, M.L. (2011). Posterior cingulate cortex: adapting behavior to a changing world. *Trends in Cognitive Sciences*, 15(4), 143–51. doi: 10.1016/j.tics.2011.02.002. 21420893 PMC3070780

[R50] Rapuano, K.M., Zieselman, A.L., Kelley, W.M., Sargent, J.D., Heatherton, T.F., Gilbert-Diamond, D. (2017). Genetic risk for obesity predicts nucleus accumbens size and responsivity to real-world food cues. *Proceedings of the National Academy of Sciences of the United States of America*, 114(1), 160–5. doi: 10.1073/pnas.1605548113. 27994159 PMC5224374

[R51] Reichelt, A.C., Morris, M.J., Westbrook, R.F. (2014). Cafeteria diet impairs expression of sensory-specific satiety and stimulus-outcome learning. *Frontiers in Psychology*, 5, 852. doi: 10.3389/fpsyg.2014.00852. PMC4146395 25221530

[R52] Richardson, N.R., Gratton, A. (1996). Behavior-relevant changes in nucleus accumbens dopamine transmission elicited by food reinforcement: an electrochemical study in rat. *The Journal of Neuroscience*, 16(24), 8160–9. 8987841 10.1523/JNEUROSCI.16-24-08160.1996 PMC6579233

[R53] Robinson, T.E., Berridge, K.C. (1993). The neural basis of drug craving: an incentive-sensitization theory of addiction. *Brain Research Reviews*, 18(3), 247–91. doi: 10.1016/0165-0173(93)90013-P. 8401595

[R54] Rothemund, Y., Preuschhof, C., Bohner, G., et al. (2007). Differential activation of the dorsal striatum by high-calorie visual food stimuli in obese individuals. *Neuroimage*, 37(2), 410–21. doi: 10.1016/j.neuroimage.2007.05.008. 17566768

[R55] Schultz, W. (1998). Predictive reward signal of dopamine neurons. *Journal of Neurophysiology*, 80(1), 1–27. doi: 10.1152/jn.1998.80.1.1. 9658025

[R56] Sclafani, A. (2001). Post-ingestive positive controls of ingestive behavior. *Appetite*, 36(1), 79–83. doi: 10.1006/appe.2000.0370. 11161347

[R57] Shearrer, G.E., Stice, E., Burger, K.S. (2018). Adolescents at high risk of obesity show greater striatal response to increased sugar content in milkshakes. *The American Journal of Clinical Nutrition*, 107(6), 859–66. doi: 10.1093/ajcn/nqy050. 29771283 PMC6037118

[R58] Small, D.M. (2010). Taste representation in the human insula. *Brain Structure and Function*, 214(5–6), 551–61. doi: 10.1007/s00429-010-0266-9. 20512366

[R59] Stice, E., Spoor, S., Bohon, C., Veldhuizen, M.G., Small, D.M. (2008). Relation of reward from food intake and anticipated food intake to obesity: a functional magnetic resonance imaging study. *Journal of Abnormal Psychology*, 117(4), 924–35. doi: 10.1037/a0013600. 19025237 PMC2681092

[R60] Stice, E., Yokum, S., Burger, K.S., Epstein, L.H., Small, D.M. (2011). Youth at risk for obesity show greater activation of striatal and somatosensory regions to food. *The Journal of Neuroscience*, 31(12), 4360–6. doi: 10.1523/JNEUROSCI.6604-10.2011. 21430137 PMC3260083

[R0060a] Stice, E., Burger, K. S., and Yokum, S. (2015). Reward region responsivity predicts future weight gain and moderating effects of the TaqIA allele. *Journal of Neuroscience*, 35(28), 10316–10324. doi: 10.1523/JNEUROSCI.6604-10.2011. 26180206 PMC4502268

[R61] Stice, E., Yokum, S. (2016). Gain in body fat is associated with increased striatal response to palatable food cues, whereas body fat stability is associated with decreased striatal response. *The Journal of Neuroscience*, 36(26), 6949–56. doi: 10.1523/JNEUROSCI.4365-15.2016. 27358453 PMC4926241

[R62] Stunkard, A.J., Messick, S. (1985). The three-factor eating questionnaire to measure dietary restraint, disinhibition and hunger. *Journal of Psychosomatic Research*, 29(1), 71–83. doi: 10.1016/0022-3999(85)90010-8. 3981480

[R63] Talepasand, S., Golzari, M. (2018). Attention control in presence of food cues in restrained and unrestrained eaters. *Noro Psikiyatri Arsivi*, 55(4), 301–6. doi: 10.5152/npa.2017.19323. 30622384 PMC6300831

[R64] Thesen, S., Heid, O., Mueller, E., Schad, L.R. (2000). Prospective acquisition correction for head motion with image-based tracking for real-time fMRI. *Magnetic Resonance in Medicine*, 44(3), 457–65. doi: 10.1002/1522-2594(200009)44:3<457::aid-mrm17>3.0.co;2-r. 10975899

[R65] Uddin, L.Q., Nomi, J.S., Hébert-Seropian, B., Ghaziri, J., Boucher, O. (2017). Structure and function of the human insula. *Journal of Clinical Neurophysiology*, 34(4), 300–6. doi: 10.1097/WNP.0000000000000377. 28644199 PMC6032992

[R66] Whitaker, K.L., Jarvis, M.J., Beeken, R.J., Boniface, D., Wardle, J. (2010). Comparing maternal and paternal intergenerational transmission of obesity risk in a large population-based sample. *The American Journal of Clinical Nutrition*, 91(6), 1560–7. doi: 10.3945/ajcn.2009.28838. 20375189

[R67] Worsley, K.J., Friston, K.J. (1995). Analysis of fMRI time-series revisited—again. *Neuroimage*, 2(3), 173–81. doi: 10.1006/nimg.1995.1023. 9343600

[R68] Yokum, S., Ng, J., Stice, E. (2011). Attentional bias to food images associated with elevated weight and future weight gain: an fMRI study. *Obesity*, 19(9), 1775–83. doi: 10.1038/oby.2011.168. 21681221 PMC4007087

