# Supplementary material for: Correlates of neural adaptation to food cues and taste: the role of obesity risk factors
Source: Soc Cogn Affect Neurosci. 2021 Mar 3;18(1):nsab018. doi: 10.1093/scan/nsab018 (PMC10074771; doi:10.1093/scan/nsab018)
Supplement: nsab018_Supp [file nsab018_supp.zip › scan-20-067-File002.docx]

**SUPPLEMENTAL MATERIALS**

**Methods**

*Substance Use and Negative Consequences Questionnaire*

Adolescents who engage in high-risk behaviors such as drug/alcohol use show differences in reward responsiveness from those who do not engage in high-risk behaviors. To adjust for possible differences, illicit drug use was measured using a self-report questionnaire assessing alcohol and drug use and negative consequences. Participants reported the frequency of use within the past year for beer and wine, hard liquor, cigarettes, marijuana, stimulants, depressants, inhalants, and hallucinogens. Additionally, participants reported the frequency of negative consequences from drug/alcohol use in the past year. Examples of negative consequences assessed include “complaints from your family or friends”, “trouble at school or work”, and “an accident or injury”.

*Robustness Check for Familial Obesity Risk Difference in Right Caudate Response*

We examined the robustness of the effect of familial obesity risk on right caudate response by excluding two potential outliers from the high risk group (see Figure 5). Subjects 55 and 138 were excluded from the sample (mean parameter estimate of right caudate cluster: 55 = 8.33; 138 = 19.83) and a t-test comparing mean parameter estimate of the right caudate cluster between the high and low familial obesity risk groups.

**Results**

*Robustness Check for Familial Obesity Risk Difference in Right Caudate Response*

When two possible outliers were removed from the sample, there was a statistically significant difference in right caudate response between the high and low familial obesity risk groups (t = -4.326, df = 162.16, p-value = 0.00002646), suggesting that the difference between the groups observed in the full sample is not driven by outliers.

**Table S1. Correlations between Pre/Post Scan Change in Liking, Wanting, and Hunger and Participant Characteristics**

|  | **Liking**^a^ | | **Wanting**^a^ | | **Hunger**^a^ | |
| --- | --- | --- | --- | --- | --- | --- |
|  | *Pearson’s r* | *p-value* | *Pearson’s r* | *p-value* | *Pearson’s r* | *p-value* |
| BMI | 0.02 | 0.81 | 0.02 | 0.77 | 0.05 | 0.58 |
| BMI-percentile | -0.01 | 0.94 | 0.02 | 0.81 | 0.11 | 0.21 |
| Age | -0.01 | 0.91 | 0.03 | 0.70 | -0.09 | 0.30 |
| TFEQ - Restraint | -0.09 | 0.32 | -0.09 | 0.29 | -0.08 | 0.33 |
| TFEQ - Disinhibition | -0.08 | 0.35 | -0.04 | 0.62 | -0.04 | 0.62 |
| BIS | 0.04 | 0.64 | 0.01 | 0.89 | 0.16 | 0.06 |
| BAS | 0.01 | 0.92 | 0.11 | 0.20 | 0.01 | 0.92 |
| Liking | -- | -- | -0.01 | 0.87 | -0.18 | 0.031* |
| Wanting | -- | -- | -- | -- | 0.14 | 0.09 |

^a^ Change in pre scan and post scan ratings

***** Significant correlation was identified, but did not survive correction for multiple comparisons (adjusted p-threshold = 0.005)

**Table S2. Interaction of Group and Time on Milkshake Liking and Wanting Ratings**

|  | **Liking^a^** | | | **Wanting^a^** | | |
| --- | --- | --- | --- | --- | --- | --- |
|  | *Pre* | *Post* | *F-statistic, p-value* | *Pre* | *Post* | *F-statistic, p-value* |
| **Familial Obesity Risk** |  |  | F= 3.32, *p=* 0.069 |  |  | F= 0.20, *p=* 0.66 |
| *High (n=118)* | 80.3 | 74.1 |  | 76.7 | 37.1 |  |
| *Low (n=36)* | 79.1 | 65.4 |  | 79.6 | 38.0 |  |
| **Drug Use – Ever in Past Year** | | | F= 0.14, *p=* 0.71 |  |  | F= 0.0, *p=* 0.997 |
| *Yes (n=49)* | 78.5 | 69.7 |  | 75.8 | 35.8 |  |
| *No (n=105)* | 80.7 | 73.3 |  | 78.1 | 38.1 |  |

^a^ Mean ± SD reported for pre and post scan

**Table S3. Correlations between Magnitude of Change in Brain Response over Time and Participant Characteristics**

|  | **Left Caudate**^a^ | | **Right Caudate**^a^ | | **Left PCC**^a,b^ | |
| --- | --- | --- | --- | --- | --- | --- |
|  | *Pearson’s r* | *p-value* | *Pearson’s r* | *p-value* | *Pearson’s r* | *p-value* |
| **BMI** | 0.10 | 0.21 | 0.07 | 0.38 | 0.17 | 0.032* |
| **BMI-percentile** | 0.12 | 0.14 | 0.08 | 0.30 | 0.18 | 0.024* |
| **%Body Fat** | 0.02 | 0.82 | 0.00 | 0.95 | 0.08 | 0.34 |
| **Age** | -0.06 | 0.44 | 0.03 | 0.75 | - 0.03 | 0.71 |
| **TFEQ - Restraint** | - 0.09 | 0.28 | 0.02 | 0.80 | - 0.24 | 0.003* |
| **TFEQ - Disinhibition** | -0.07 | 0.40 | -0.13 | 0.11 | -0.15 | 0.07 |
| **BIS** | -0.02 | 0.80 | 0.16 | 0.05 | 0.03 | 0.74 |
| **BAS** | 0.01 | 0.91 | 0.01 | 0.94 | 0.02 | 0.82 |
|  | **Right PCC**^a,b^ | | **Left OSC**^c^ | | **Right OSC**^c^ | |
|  | *Pearson’s r* | *p-value* | *Pearson’s r* | *p-value* | *Pearson’s r* | *p-value* |
| **BMI** | 0.05 | 0.53 | 0.01 | 0.86 | 0.12 | 0.14 |
| **BMI-percentile** | 0.09 | 0.27 | 0.00 | 0.99 | 0.08 | 0.31 |
| **%Body Fat** | 0.00 | 0.96 | -0.05 | 0.53 | -0.02 | 0.80 |
| **Age** | -0.06 | 0.49 | 0.00 | 0.95 | 0.03 | 0.68 |
| **TFEQ - Restraint** | -0.05 | 0.55 | -0.05 | 0.57 | -0.07 | 0.37 |
| **TFEQ - Disinhibition** | -0.09 | 0.26 | -0.01 | 0.88 | -0.07 | 0.42 |
| **BIS** | 0.06 | 0.45 | 0.01 | 0.86 | -0.05 | 0.58 |
| **BAS** | 0.01 | 0.94 | 0.07 | 0.38 | 0.12 | 0.16 |

**^a^** Change from early exposure to late exposure to milkshake cue

**^b^** PCC: posterior cingulate cortex

**^c^** Change from early exposure to late exposure to milkshake taste; OSC: oral somatosensory cortex

***** Significant correlation was identified with uncorrected p, but did not survive correction for multiple comparisons

**Table S4.** **Interaction of Group and Time on Brain Response to Milkshake Cue and Receipt in Regions of Interest**

|  | **Left Caudate**^a^ | | | **Right Caudate**^a^ | | |
| --- | --- | --- | --- | --- | --- | --- |
| **Familial Obesity Risk** | *Early* | *Late* | *ANOVA* | *Early* | *Late* | *ANOVA* |
| *High* | 0.06 | 0.88 | F= 0.71, *p=* 0.40 | -0.09 | -0.29 | F= 0.24, *p=* 0.62 |
| *Low* | -0.75 | -1.26 |  | -2.1 | -1.6 |  |
| **Drug Use – Ever in Past Year** | *Early* | *Late* | *ANOVA* | *Early* | *Late* | *ANOVA* |
| *Yes* | -0.27 | 0.21 | F= 0.001, *p=* 0.98 | -1.46 | -1.41 | F= 0.006, *p=* 0.94 |
| *No* | -0.07 | 0.46 |  | -0.14 | -0.21 |  |
|  | **Left PCC**^a,b^ | | | **Right PCC**^a,b^ | | |
| **Familial Obesity Risk** | *Early* | *Late* | *ANOVA* | *Early* | *Late* | *ANOVA* |
| *High* | -0.12 | 0.67 | F= 0.12, *p=* 0.73 | -0.11 | 1.28 | F= 0.27, *p=* 0.60 |
| *Low* | -0.75 | 0.38 |  | -0.86 | -0.08 |  |
| **Drug Use – Ever in Past Year** | *Early* | *Late* | *ANOVA* | *Early* | *Late* | *ANOVA* |
| *Yes* | -0.91 | 0.29 | F= 0.29, *p=* 0.59 | -0.87 | 0.14 | F= 0.10, *p=* 0.76 |
| *No* | 0.03 | 0.74 |  | -0.01 | 1.34 |  |
|  | **Left OSC**^c^ | | | **Right OSC**^c^ | | |
| **Familial Obesity Risk** | *Early* | *Late* | *ANOVA* | *Early* | *Late* | *ANOVA* |
| *High* | -15.5 | 25.5 | F= 0.61, *p=* 0.43 | -11.4 | 27.2 | F= 0.15, *p=* 0.70 |
| *Low* | -14.0 | 16.9 |  | -16.0 | 18.5 |  |
| **Drug Use – Ever in Past Year** | *Early* | *Late* | *ANOVA* | *Early* | *Late* | *ANOVA* |
| *Yes* | -24.4 | 21.8 | F= 0.87, *p=* 0.35 | -20.4 | 24.3 | F= 1.06, *p=* 0.30 |
| *No* | -10.7 | 24.5 |  | -8.77 | 25.6 |  |

**^a^** Response to milkshake cue

**^b^** PCC: posterior cingulate cortex

**^c^** Response to milkshake taste; OSC: oral somatosensory cortex

**Table S5. Correlations between Magnitude of Change in Brain Response over Time and Liking, Wanting, and Hunger**

|  | **Left Caudate**^a^ | | **Right Caudate**^a^ | | **Left PCC**^a,b^ | |
| --- | --- | --- | --- | --- | --- | --- |
|  | *Pearson’s r* | *p-value* | *Pearson’s r* | *p-value* | *Pearson’s r* | *p-value* |
| **Liking** | 0.00 | 0.98 | -0.03 | 0.70 | -0.06 | 0.43 |
| **Wanting** | 0.04 | 0.63 | -0.03 | 0.75 | -0.04 | 0.64 |
| **Hunger** | 0.03 | 0.69 | 0.10 | 0.23 | 0.10 | 0.20 |
|  | **Right PCC**^a,b^ | | **Left OSC**^c^ | | **Right OSC**^c^ | |
|  | *Pearson’s r* | *p-value* | *Pearson’s r* | *p-value* | *Pearson’s r* | *p-value* |
| **Liking** | 0.06 | 0.47 | -0.15 | 0.08 | -0.05 | 0.56 |
| **Wanting** | 0.00 | 0.96 | 0.03 | 0.74 | 0.08 | 0.31 |
| **Hunger** | 0.06 | 0.46 | 0.05 | 0.55 | 0.08 | 0.34 |

**^a^** Change from early exposure to late exposure to milkshake cue

**^b^** PCC: posterior cingulate cortex

**^c^** Change from early exposure to late exposure to milkshake taste; OSC: oral somatosensory cortex

**Table S6. Average BOLD Response to Milkshake Cue and Receipt**

| **Milkshake Cue > Tasteless Cue** | | **x^a^** | **y** | **z** | ***k^b^*** | **z-value** | **p_FWE_** |
| --- | --- | --- | --- | --- | --- | --- | --- |
|  | Right Caudate | 9 | 15 | 3 | 543 | 7.02 | < 0.001 |
|  | Left Accumbens/Caudate | -6 | 6 | -3 |  | 7.01 | < 0.001 |
|  | Left Caudate | -6 | 9 | 6 |  | 6.27 | < 0.001 |
|  | Left Occipital Fusiform Gyrus | -21 | -84 | -12 | 12 | 5.85 | < 0.001 |
|  | Left Anterior Cingulate Cortex | -3 | 30 | 18 | 50 | 5.29 | 0.002 |
|  | Left Anterior Cingulate Cortex | -3 | 36 | 6 |  | 5.27 | 0.002 |
|  | Left Anterior Cingulate Cortex | 3 | 30 | 24 |  | 5.00 | 0.008 |
| **Milkshake Receipt > Tasteless Receipt** | |  |  |  |  |  |  |
|  | Right Precentral Gyrus | 42 | -12 | 33 | 4754 | > 8.0 | < 0.001 |
|  | Left Precentral Gyrus | -39 | -15 | 33 |  | > 8.0 | < 0.001 |
|  | Left Postcentral Gyrus | -48 | -15 | 39 |  | > 8.0 | < 0.001 |
|  | Left Cerebellum VI | -15 | -63 | -21 | 945 | > 8.0 | < 0.001 |
|  | Right Cerebellum VI | 15 | -63 | -21 |  | > 8.0 | < 0.001 |
|  | Left Intracalcarine Cortex | -12 | -78 | 6 |  | 7.30 | < 0.001 |
|  | Right Precentral Gyrus | 21 | -27 | 60 | 41 | > 8.0 | < 0.001 |
|  | Left Precentral Gyrus | -21 | -30 | 60 | 39 | > 8.0 | < 0.001 |
|  | Right Supplementary Motor Cortex | 3 | -6 | 63 | 232 | > 8.0 | < 0.001 |
|  | Right Supplementary Motor Cortex/Anterior Cingulate Gyrus | 6 | 3 | 48 |  | 7.77 | < 0.001 |
|  | Right Precuneus | 27 | -45 | 18 | 25 | 5.45 | 0.001 |
|  | Right Precuneus | 21 | -39 | 24 |  | 5.07 | 0.006 |
|  | Right Superior Frontal Gyrus | 21 | -9 | 66 | 10 | 5.28 | 0.002 |
| ^a^ MNI coordinates (mm)  ^b^ Cluster size | | | | | | | |
